# Rifaximin versus Nonabsorbable Disaccharides for the Treatment of Hepatic Encephalopathy: A Meta-Analysis

**DOI:** 10.1155/2013/236963

**Published:** 2013-04-03

**Authors:** Dong Wu, Shu-Mei Wu, Jie Lu, Ying-Qun Zhou, Ling Xu, Chuan-Yong Guo

**Affiliations:** Department of Gastroenterology, Shanghai tenth People's Hospital, Tongji University School of Medicine, Shanghai 200072, China

## Abstract

*Background*. Many studies have found that the antibiotic rifaximin is effective for the treatment of hepatic encephalopathy. However, there is no uniform view on the efficacy and safety of rifaximin. *Methods*. We performed a meta-analysis through electronic searches to evaluate the efficacy and safety of rifaximin in comparison with nonabsorbable disaccharides. *Results*. A total of 8 randomized controlled trials including 407 patients were included. The efficacy of rifaximin was equivalent to nonabsorbable disaccharides according to the statistical data (risk ratio (RR): 1.06, 95% CI: 0.94–1.19; *P* = 0.34). Analysis showed that patients treated with rifaximin had better results in serum ammonia levels (weighted mean difference (WMD): −10.63, 95% CI: −30.63–9.38; *P* = 0.30), mental status (WMD: −0.32, 95% CI: −0.67–0.03; *P* = 0.07), asterixis (WMD: −0.12, 95% CI: −0.31–0.08; *P* = 0.23), electroencephalogram response (WMD: −0.21, 95% CI: −0.34–−0.09; *P* = 0.0007), and grades of portosystemic encephalopathy (WMD: −2.30, 95% CI: −2.78–−1.82; *P* < 0.00001), but only the last ones had statistical significance. The safety of rifaximin was better than nonabsorbable disaccharides (RR: 0.19, 95% CI: 0.10–0.34; *P* < 0.00001). *Conclusion*. Rifaximin is at least as effective as nonabsorbable disaccharides, maybe better for the treatment of hepatic encephalopathy. And the safety of rifaximin is better.

## 1. Introduction

Hepatic encephalopathy (HE) is a complex and reversible neuropsychiatric syndrome that results from acute or chronic liver diseases, such as hepatic cirrhosis, alcoholic liver disease [1]. HE is an important complication of hepatic cirrhosis and is an independent predictor of mortality in patients with cirrhosis [2]. HE occurs in the presence of insufficient hepatic clearance of toxins absorbed from the intestine resulting in neurochemical abnormalities across the blood brain barrier [3]. The symptoms of HE, manifested on a continuum, are deterioration in mental status, with psychomotor dysfunction, impaired memory, increased reaction time, sensory abnormalities, poor concentration, disorientation, even coma, and death [4, 5]. Overt HE means high mortality and poor prognosis. 1-year mortality for patients with severe HE in ICU is 54% [6]. Episodes of overt HE result in frequent hospitalizations and pose a formidable burden on the healthcare system, especially in China, a developing country with 100000000 hepatitis B carriers [7].

 Diagnosis of overt hepatic encephalopathy should be made after the exclusion of other brain disorders [8] and based on two types of symptoms. Impaired mental status, as defined by the Conn score, with higher scores indicating more severe impairment, and impaired neuromotor function includes hyperreflexia, rigidity, myoclonus, and asterixi [9, 10]. Minimal hepatic encephalopathy (MHE) that may has no clinical manifestations could be detected only by neuropsychological methods include portosystemic encephalopathy (PSE) syndrome test, Psychometric-Hepatic-Encephalopathy-Sum- (PHES-) Score [11]. Elevated serum ammonia level is an effective index of HE and is detected in 60%–80% of affected patients, but a single ammonia level in the diagnosis of HE is uncertain given the substantial overlap of ammonia levels in both patients with and without encephalopathy [12]. Current treatment strategies include measures aimed at reducing the serum level of ammonia, providing specialized nursing care as well as correcting precipitating factors such as gastrointestinal hemorrhage, infection, constipation, and electrolyte disturbances [5].

 Up to now, nonabsorbable disaccharides such as lactulose (*β*-galactoside fructose) and lactitol (*β*-galactoside sorbitol) have been the first-line drug for the treatment of HE. They are directed at reducing the serum level of ammonia, since they decrease the absorption of ammonia through cathartic effects and by altering the colonic pH [13]. The side effects of nonabsorbable disaccharides include abdominal pain, flatulence, and severe diarrhea, which may lead to the cessation of therapy [14]. Antibiotics such as neomycin, vancomycin, metronidazole, and rifaximin were shown to be effective in the treatment of both acute and chronic encephalopathy. They reduce bacterial production of ammonia through suppression of intestinal flora [15, 16]. Due to serious side effects such as ototoxicity and nephrotoxicity, most antibiotics exception of rifaximin are not suitable for long-term use for the treatment of HE [17]. Rifaximin is a minimally absorbed oral gastrointestinal selective antibiotic, with very few systemic side effects and has a low risk of inducing bacterial resistance [18]. Some studies showed that rifaximin is superior to lactulose and antimicrobials in patients with mild-to-moderate severe HE [19], but a larger meta-analysis including twelve studies comparing rifaximin to conventional oral therapy showed no significant difference between the two interventions [20]. Previous studies have reached different conclusions, therefore, we conducted a meta-analysis to evaluate all RCTs comparing rifaximin to nonabsorbable disaccharides for the treatment of patients with HE.

## 2. Methods

### 2.1. Search Strategy and Study Selection

The Pubmed, EMbase, Cochrane Library, EMBASE, CINAHL, and Science Citation Index (ISI Web of Science) were searched to collect all randomized controlled trials comparing rifaximin to nonabsorbable disaccharides for the treatment of hepatic encephalopathy (last search update: 20th August 2012) without language restriction.

Database specific search terms for rifaximin (rifaximin, rifamycins), disaccharides (disaccharides, lactulose, lactitol, and sugar alcohols) were combined by limiting the searches to studies of human patients and reports of clinical trials. All reference lists of eligible studies were hand-searched to avoid missing any relevant studies. Two reviewers (Dong Wu and Shu-Mei Wu) independently assessed the eligibility of all potential abstracts and titles. 

### 2.2. Inclusion and Exclusion Criteria

Inclusion criteria were (1) patient population over 18 years of age; (2) signs and symptoms of acute, chronic HE according to Conn's modification of Parsons Smith classification [21]. Inclusion was regardless of publication status, language. Exclusion criteria were (1) non-controlled clinical trial; (2) trials including patients and with psychiatric illness, with undercurrent infections, with hypersensitivity to rifaximin and other antibiotics and/or intolerance to nonabsorbable disaccharides; (3) studies that compared the use of rifaximin versus placebo; (4) trials that included individuals affected by gastrointestinal bleeding.

### 2.3. Definitions

Clinical efficacy was defined as improvement in the HE clinical syndrome as in passing to a lower stage or a significant decrease in the portosystemic encephalopathy index. Partial neurological response was measured by mental status scores according to Conn's classification [21, 22], and adverse events in this study were severe diarrhea, episodes of intense abdominal pain. Serum ammonia levels were assessed at the end of the treatment. The severity of asterixis was graded according to established criteria as follows: 0, no tremors; 1, few flapping motions; 2, occasional flapping motions; 3, frequent flapping motions; and 4, almost continuous flapping motions. Electroencephalogram (EEG) abnormalities recorded in patients with HE were scored according to criteria as follows: 0, normal EEG; 1, normal-limit EEG; 2, mild signs of encephalopathy; 3, distinctive features of encephalopathy; and 4, signs of severe encephalopathy. Grades of PSE were calculated as the sum of the degree of mental status abnormality scores, the severity of asterixis, level of serum ammonia, and the degree of EEG abnormality [23].

### 2.4. Data Extraction

Two authors extracted information independently, and disagreements were resolved by discussion. The following data were extracted from each included article: name of the first author, year of publication, country of origin, number of patients, daily dosage of oral therapy, duration of the treatment, allocation sequence generation, and methods used to deal with missing data. Clinical variables extracted were the effectiveness of rifaximin and nonabsorbable disaccharides, side effects, serum ammonia levels, and psychometric parameters.

### 2.5. Assessment of Methodological Quality

The quality of each study was assessed according to the Cochrane Collaboration's tool for assessing risk of bias. Each of the items in the checklist was scored as “yes”, “no”, “unclear”, or “not available” [24].

### 2.6. Statistical Analysis

Statistical analyses were performed using RevMan Version 5.0.5 software. The effect measures estimated were risk ratio (RR) for dichotomous data and weighted mean difference (WMD) for continuous data, both reported with 95% confidence intervals (CIs). Pooled RR or WMD was calculated using the general inverse variance (IV) with random effect model. The heterogeneity between studies was examined by DerSimonian and Laird (DL) Q statistical analysis. If results were heterogeneous (*P* < 0.1), random effect model was used using the DL methods. Pooled RR or WMD was presented as standard plots with 95%. 

## 3. Results

**Figure 1:**
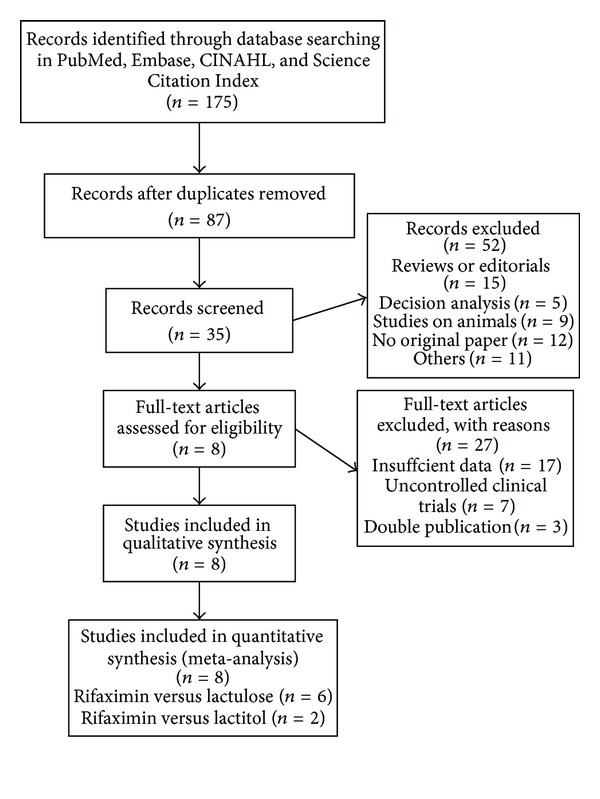
Flow diagram of included studies of meta-analysis.

A total of 175 potentially relevant references were identified with only eight being controlled clinical trials (Figure 1) [21, 25–31]. The outcomes were extracted from each trial. The main characteristics of the trials included in the meta-analysis are shown in Table 1. Most clinical trials were single-center except one, which was multicentric [25]. The dose of rifaximin was usually 1200 mg/day, and the dose of nonabsorbable disaccharides ranged from 45 to 120 mL/d for lactulose and 60 g/d for lactitol.

The quality assessments of the eight randomized controlled trials (RCTs) are shown in Table 2. The allocation system was described in five trials, and allocation concealment was defined in six trials. All the trials were blinded to patients; however, only three trials were blinded to observers. Methods for handling missing data, description of drop-outs were not described in any of the included studies.

### 3.1. Primary Outcomes

#### 3.1.1. Efficacy

**Figure 2:**
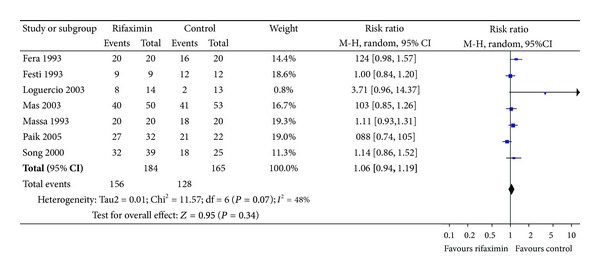
Efficacy of rifaximin versus nonabsorbable disaccharides in the treatment of hepatic encephalopathy. M-H: Mantel Haenszel.

Clinical efficacy was defined as improvement in the HE clinical syndrome as in passing to a lower stage or a significant decrease in the portosystemic encephalopathy index. HE index = (grade of mental state) × 3 + (grade of number connection test) + (grade of flapping tremor) + (grade of blood ammonia) [32]. As the RCT reported by Bucci did not mention the number of patients with clinical efficacy, we only had seven RCTs to assess the effectiveness by comparing rifaximin to nonabsorbable disaccharides. Using the random-effect model (*χ* ^2^ = 11.57, df = 6(*P* = 0.07), *I* ^2^ = 48% < 50%), the pooled analysis of seven trials that investigated the efficacy of rifaximin (*n* = 184) versus nonabsorbable disaccharides (*n* = 165) showed no significant difference (RR: 1.06, 95% CI: 0.94–1.19; *P* = 0.34) (Figure 2).

#### 3.1.2. Safety or Adverse Events

**Figure 3:**
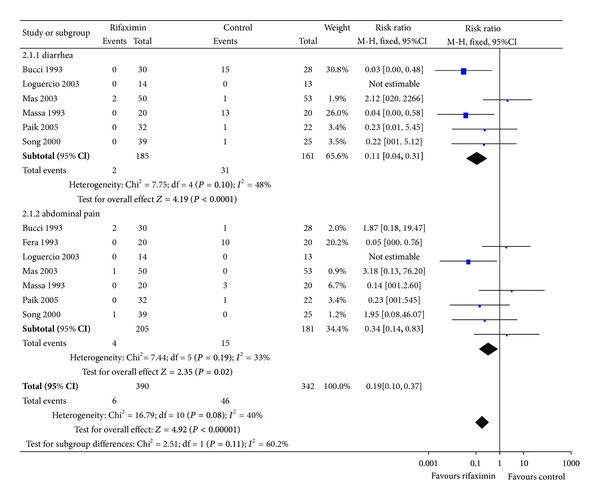
Adverse events of rifaximin versus nonabsorbable disaccharides in the treatment of hepatic encephalopathy. M-H: Mantel Haenszel.

In our meta-analysis, two common side effects were assessed: severe diarrhea and abdominal pain. The adverse events were pooled and compared between the group of rifaximin (*n* = 390) and the control group (*n* = 342). A subgroup analysis was done to compare each side effect separately. Patients in rifaximin group had less risk of suffering from diarrhea (RR: 0.11, 95% CI: 0.04–0.31; *P* < 0.0001). The rate of abdominal pain was also lower in rifaximin group (RR: 0.34, 95% CI: 0.14–0.83; *P* = 0.02). Obviously, combined analysis of two adverse events showed that rifaximin was safer than nonabsorbable disaccharides for the treatment of hepatic encephalopathy (RR: 0.19, 95% CI: 0.10–0.37; *P* < 0.00001) (Figure 3).

### 3.2. Secondary Outcomes

Psychometric parameters: there was no significant difference in the improvement in mental status and grade of asterixis (rifaximin versus control) (*P* = 0.07 and *P* = 0.23, resp.). For EEG and PSE sum, our analysis showed statistically significant difference favoring the use of rifaximin (*P* = 0.0007,  *P* < 0.00001, resp.) (Table 3).

**Figure 4:**
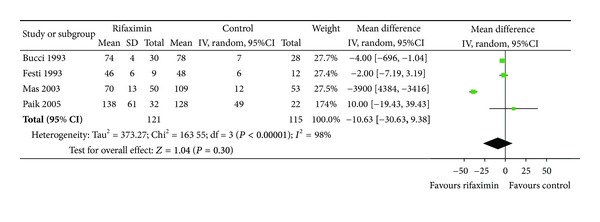
Blood ammonia levels at the end of treatment: rifaximin versus nonabsorbable disaccharides. IV: inverse variance.

Blood ammonia level: we extracted blood ammonia level at the end of 4 RCTs [21, 25, 26, 28], a significant reduction in serum ammonia level was observed in both treatment groups (rifaximin versus nonabsorbable disaccharides); however, there was no significant difference (WMD: −10.63, 95% CI: −30.63–9.38; *P* = 0.30) (Figure 4).

### 3.3. Sensitivity Analysis

The ethnical differences and the presence of acute or chronic HE were important factors that might influence the effectiveness of rifaximin; we compared clinical efficacy among patients of Europe, patients with acute or chronic HE, respectively. Sensitivity analysis showed that the ethnical differences and the presence of acute HE did not influence the efficacy of rifaximin (*P* = 0.07,  *P* = 0.70, resp.); however, significant difference in the treatment of acute HE, favoring the use of rifaximin (*P* = 0.005) (Table 4).

### 3.4. Publication Bias

**Figure 5:**
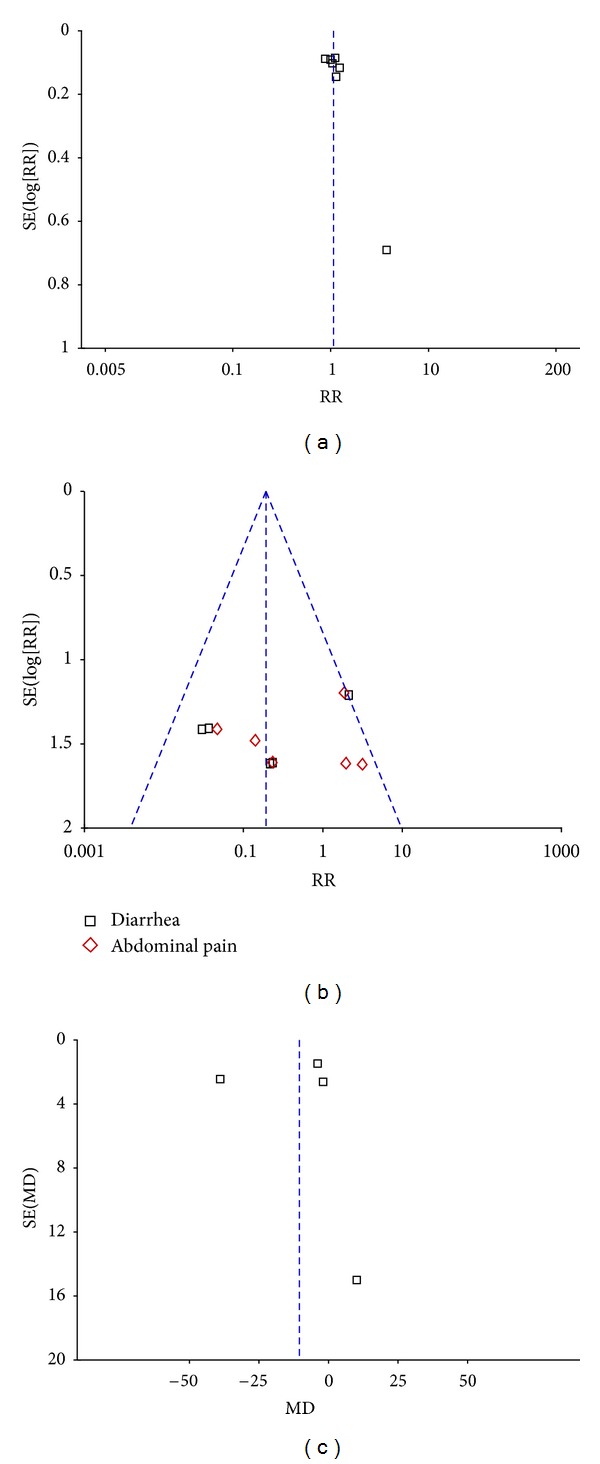
Funnel plot of meta-analysis. (a): effectiveness, (b): adeverse events and (c): blood ammonia level.

Figure 5 showed the funnel plot of meta-analysis. The points were not uniformly distributed on both sides of the longitudinal axis, suggesting the presence of publication bias.

## 4. Discussion

Chronic liver disease and cirrhosis affect hundreds of millions of patients all over the world, especially in China. One of these recurrent and difficult to treat complications is hepatic encephalopathy [33]. Overt hepatic encephalopathy affects from 30 to 45% of patients with cirrhosis, and a higher percentage may be affected by minimal hepatic encephalopathy (MHE). It is a spectrum ranging from minimal hepatic encephalopathy (MHE) without recognizable clinical symptoms to overt HE with risk of cerebral edema and death. HE that results in diminished quality of life and survival is serious challenges on the healthcare system [7]. Our treatment strategy is to reduce the production and absorption of ammonia and other gut-derived toxins. Many overt HE can be improved when precipitating factors are corrected, such as infection, gastrointestinal bleeding, dehydration, and electrolyte disturbances [5]. Nonabsorbable disaccharides (lactulose and lactitol) were considered as standard treatment for hepatic encephalopathy, that have been proved effectively for the treatment of HE. Some severe adverse events, including diarrhea, abdominal pain, vomiting, and flatulence, may lead to the cessation of therapy with disaccharides. Some minimally absorbed antibiotics, such as neomycin, vancomycin, metronidazole, and oral quinolones, were previously shown in some studies to be effective in the treatment of both acute and chronic encephalopathy [16]. The significant risk of severe toxicity is the reason why most agents are seldom used in practice. Rifaximin is a minimally absorbed antimicrobial agent with a broad spectrum against gram-positive and gram-negative aerobic and anaerobic enteric bacteria [34]. Rifaximin seems the ideal drug that appears to be effective in the treatment of HE without carrying the risk of severe side effects. In 2010, FDA approved rifaximin as a drug of HE treatment. Is it reasonable to consider rifaximin as a first-line drug for HE? There is no consensus on it. Expense of rifaximin is great, and effectiveness of rifaximin is uncertain compared to nonabsorbable disaccharides. Results of some underpowered randomized controlled trials were inconsistent.

 Here, we performed a meta-analysis containing eight randomized controlled trials assessing the efficacy and safety of rifaximin versus nonabsorbable disaccharides. Furthermore, we assessed the reduction of blood ammonia levels, and psychometric outcomes (mental status, grade of asterixis, electroencephalogram, portosystemic encephalopathy sum). Our study showed that rifaximin was as effective as nonabsorbable disaccharides but with fewer adverse events. A randomized, double-blind, placebo-controlled trial showed that rifaximin was effective in preventing hepatic encephalopathy. Over a 6-month period, treatment with rifaximin maintained remission from hepatic encephalopathy more effectively than placebo. Rifaximin treatment also significantly reduced the risk of hospitalization involving hepatic encephalopathy [35]. So, rifaximin is effective in the treatment and prevention of hepatic encephalopathy, but more studies are needed to assess its safety, including tolerance, toxicity, bacterial resistance, and mycotic infection.

For the secondary outcomes, patients in rifaximin group had lower serum ammonia levels, superior mental status, and asterixis profiles versus the control group with no statistical significance. On the other hand, the grade of EEG and PSE sums showed better results for patients in rifaximin group, when compared to their controls. So, maybe we can build an accurate scoring criteria to assess and quantify subtle clinical changes in the treatment of HE. Of course, there were some limitations in our meta-analysis. The RCTs included in our study did not have enough number of cases. We need more RCTs in recent years to make the conclusion more convincing. Five of the eight RCTs were carried out in Italy, and there were little information about other countries. These reasons will lead to bias. So, further studies on larger populations of patients are necessary to obtain more sufficient evidence for the evaluation of rifaximin versus nonabsorbable disaccharides for HE.

In summary, this study shows that rifaximin is as effective as nonabsorbable disaccharides, maybe better in some psychometric outcomes, with fewer adverse events. Sensitivity analysis showed significant difference in the treatment of acute HE, favoring the use of rifaximin, but the result may be not credible because of small samples. We suggest that rifaxinmin should be used as second-line, because of its expensive price and safety in long-term use. Patients who have severe adverse events in disaccharides therapy could use rifaximin instead.

## Figures and Tables

**Table 1:** RCTs with rifaximin (1200 mg/day) in the treatment of hepatic encephalopathy.

Authors	Country	Year	Rifaximi (*n*)	Control (*n*)	Comparative agent	Duration of treatment	Outcomes
Paik et al. [21]	South Korea	2005	32	22	Lactulose 90 mL/d	7 d	Mental status, flapping tremors, NCT, blood ammonia, and HE index
Verhagen et al. [24]	Italy	2001	9	12	Lactulose 60 mL/d	21 d	Asterixis, EEG, blood ammonia
Festi et al. [25]	Italy	1993	30	28	Lactulose 45 mL/d	15 d	Mental status, asterixis, cancellation test, Reitan test, EEG, and blood ammonia
Bucci and Palmieri [26]	Italy	1993	20	20	Lactulose 90 mL/d	15 d	Mental status, asterixis, cancellation test, EEG, trail making test, PSE index, and blood ammonia
Massa et al. [27]	Spain	1993	50	53	Lactitol 60 g/d	5–10 d	HE grade, mental status, asterixis, NCT, EEG, PSE index, and blood ammonia
Mas et al. [28]	Italy	2003	14	13	Lactulose 90 g/d	3 month	Mental status, asterixis, NCT, and blood ammonia
Loguercio et al. [29]	Italy	2003	20	20	Lactulose 120 mL/d	3 month	Mental status, asterixis, cancellation test, Reitan test, EEG, and PSE severity
Fera et al. [30]	South Korea	1993	39	25	Lactulose 90 mL/d	7 d	Blood ammonia, mental status, flapping tremors, NCT, and HE index

NCT: number connection test, EEG: electroencephalogram, and PSE: portosystemic encephalopathy.

**Table 2:** Summary of methodological quality of included studies on the basis of review authors' judgments.

Included studies	Allocation system	Allocation concealment	Patient	Blind observer	Assessor	Handling of missing data
Paik et al. 2005 [21]	Yes	Yes	Yes	No	NA	Unclear
Festi et al. 1993 [25]	No	No	Yes	No	NA	Unclear
Bucci and Palmieri 1993 [26]	Yes	Yes	Yes	Yes	NA	Unlcear
Massa et al. 1993 [27]	No	Yes	Yes	Yes	NA	Unclear
Mas et al. 2003 [28]	Yes	Yes	Yes	Yes	NA	Unclear
Loguercio et al. 2003 [29]	No	No	Yes	No	NA	Unclear
Fera et al. 1993 [30]	Yes	Yes	Yes	No	NA	Unclear
Song et al. 2000 [31]	Yes	Yes	Yes	No	NA	Unclear

NA: not available.

**Table 3:** Meta-analysis of psychchometric outcomes between rifaximin and nonabsorbable disaccharides.

Psychometric outcomes	Rifaximin			Control			Mean difference IV, random, 95% CI
	Mean	SD	Total	Mean	SD	Total
Mental status							

Bucci and Palmieri 1993 [26]	0.8	0.5	30	1.2	0.3	28	−0.40 [−0.61, −0.19]
Loguercio et al. 2003 [29]	0.42	0.67	14	0.9	0.74	13	−0.48 [−1.01, 0.05]
Massa et al. 1993 [27]	0.6	0.2	20	1.2	0.3	20	−0.60 [−0.76, −0.44]
Paik et al. 2005 [21]	0.5	0.7	32	0.3	0.4	22	0.2 [−0.09, 0.49]

Total (95% CI)			96			83	−0.32 [−0.67, 0.03]

Heterogeneity: Τ^²^ = 0.10; *χ* ^2^ = 22.09; df = 3 (*P* < 0.0001); *I* ^2^ = 86%							
Test for overall effect: *Z* = 1.80 (*P* = 0.07)							

Asterixis							

Bucci and Palmieri 1993 [26]	0.5	0.3	30	1.2	0.3	28	−0.40 [−0.61, −0.19]
Mas et al. 2003 [28]	0	0.5	50	0	0.5	53	0.00 [−0.19, 0.19]
Massa et al. 1993 [27]	0.1	0.2	20	0.1	0.2	20	0.00 [−0.12, 0.12]
Paik et al. 2005 [21]	0.3	0.7	32	0.4	0.6	22	−0.10 [−0.45, 0.25]

Total (95% CI)			132			123	−0.12 [−0.31, 0.08]

Heterogeneity: Τ^²^ = 0.03; *χ* ^2^ = 10.85; df = 3 (*P* = 0.01); *I* ^2^ = 72%							
Test for overall effect: *Z* = 1.19 (*P* = 0.23)							

EEG							

Bucci and Palmieri 1993 [26]	0.4	0.2	30	0.6	0.3	28	−0.20 [−0.33, −0.07]
Mas et al. 2003 [28]	0.6	0.9	50	0.9	0.9	53	−0.30 [−0.65, 0.05]

Total (95% CI)			80			81	−0.21 [−0.34, −0.09]

Heterogeneity: Τ^²^ = 0.00; *χ* ^2^ = 0.28; df = 1 (*P* = 0.60); *I* ^2^ = 0%							
Test for overall effect: *Z* = 3.37 (*P* = 0.0007)							

PSE sum							

Mas et al. 2003 [28]	4	0.1	50	6	2	53	−2.00 [−2.54, −1.46]
Massa et al. 1993 [27]	3	0.5	20	5.5	0.5	20	−2.50 [−2.78, −1.82]

Total (95% CI)			70			73	−2.30 [−2.78, −1.82]

Heterogeneity: Τ^²^ = 0.07; *χ* ^2^ = 2.48; df = 1 (*P* = 0.12); *I* ^2^ = 60%							
Test for overall effect: *Z* = 9.40 (*P* < 0.00001)							

IV: inverse variance, EEG: electroencephalogram, and PSE: portosystemic encephalopathy.

**Table 4:** Result of sensitivity analysis.

Variable	Patients (rifaximin/control)	Pooled RR	*P*
All in Europe [24–29]	231 (113/118)	3.16 [0.92, 10.93]	0.07
Acute HE [21, 27, 30]	157 (82/75)	0.77 [0.20, 2.93]	0.70
Chronic HE [26, 28, 29]	107 (54/53)	7.6 [1.87, 30.78]	0.005

HE: hepatic encephalopathy; control lactulose or lactitol; RR: relative risk.
